# Laser fabrication of MXene-based planar micro-supercapacitors

**DOI:** 10.3389/fchem.2025.1676794

**Published:** 2025-09-02

**Authors:** Qian-Kun Li, Jin-Yong Qi, Bo Li, Ke-Xue Li, Feng-Yuan Lin, Ji-Long Tang, Zhi-Peng Wei

**Affiliations:** State Key Laboratory of High Power Semiconductor Lasers, School of Physics, Changchun University of Science and Technology, Changchun, China

**Keywords:** MXene, planar supercapacitors, laser ablation, laser cutting, laser modification

## Abstract

This mini-review summarizes recent advances in MXene-based planar micro-supercapacitors fabricated by laser technologies. Laser ablation enables ultrafast maskless patterning, laser cutting achieves mechanically adaptive flexible arrays, and laser modification optimizes microstructure and surface chemistry. Representative studies, performance correlations, and prospects for scalable, high-energy-density MSCs are highlighted.

## 1 Introduction

MXenes are two-dimensional transition-metal carbides, nitrides, and carbonitrides (Dey et al., 2023; Huang et al., 2024; Rasheed et al., 2025). Their metallic conductivity, tunable surface chemistry, and abundant interlayer ion-transport channels make them highly attractive for electrochemical energy storage (Ali et al., 2025; Ponnalagar et al., 2024; Zheng et al., 2022). Their high theoretical volumetric capacitance and capability for rapid surface redox reactions make them highly promising electrode materials for supercapacitors (Bi W. C. et al., 2024; Khan et al., 2024; Nouseen and Pumera, 2025). Compared with traditional carbon-based materials, MXenes offer superior rate performance and energy density while maintaining mechanical flexibility, which is essential for emerging micro-scale energy storage applications (Bi S. et al., 2024; Dananjaya et al., 2025; Elugoke et al., 2024).

Micro-supercapacitors (MSCs) are compact energy storage devices characterized by ultrafast charge-discharge rates, long cycling stability, and seamless integration with on-chip and wearable electronics (Huang et al., 2024; Li et al., 2025; Patil et al., 2024). Typically, MSCs adopt planar interdigitated electrode architectures, which maximize the active surface area and enable efficient ion diffusion (Fu et al., 2024; Han et al., 2023b; Pham and Huynh, 2024). This geometry also supports series and parallel modular configurations, enabling flexible voltage and capacity tuning while ensuring compatibility with miniaturized and flexible electronic systems (Fu et al., 2020; Hussain et al., 2024; Zhou et al., 2023).

Laser-based fabrication technologies provide an effective pathway to overcome the challenges of scalable MSC manufacturing by offering maskless, rapid, and high-precision patterning compatible with diverse substrates (Devi et al., 2023; Han et al., 2023a; Han et al., 2025; Li et al., 2023; You et al., 2020). In this mini-review, we comprehensively summarize recent advances in the fabrication of MXene-based planar MSCs using laser technologies. This review discusses three laser-based strategies, including laser ablation, laser cutting, and laser modification. Representative studies are highlighted to illustrate the connection between processing methods, device architectures, and electrochemical performance. Finally, current challenges and future prospects for scalable, multifunctional, and high-energy-density MXene-based MSCs fabricated via laser technologies are discussed.

## 2 Laser ablation technologies

Laser ablation is a widely adopted and efficient strategy for fabricating MXene-based planar micro-supercapacitors (MSCs). The general approach involves first depositing an MXene film on a target substrate such as glass, PET, or Si/SiO_2_ wafers, followed by direct laser ablation to form interdigitated electrode patterns. During this process, the laser selectively removes predefined areas of the MXene film, leaving conductive paths that simultaneously function as both the active material and the current collector. This maskless, non-contact method eliminates the need for complex lithographic steps or metal electrodes, enabling rapid, low-cost, and highly precise patterning suitable for on-chip integration.

**FIGURE 1:**
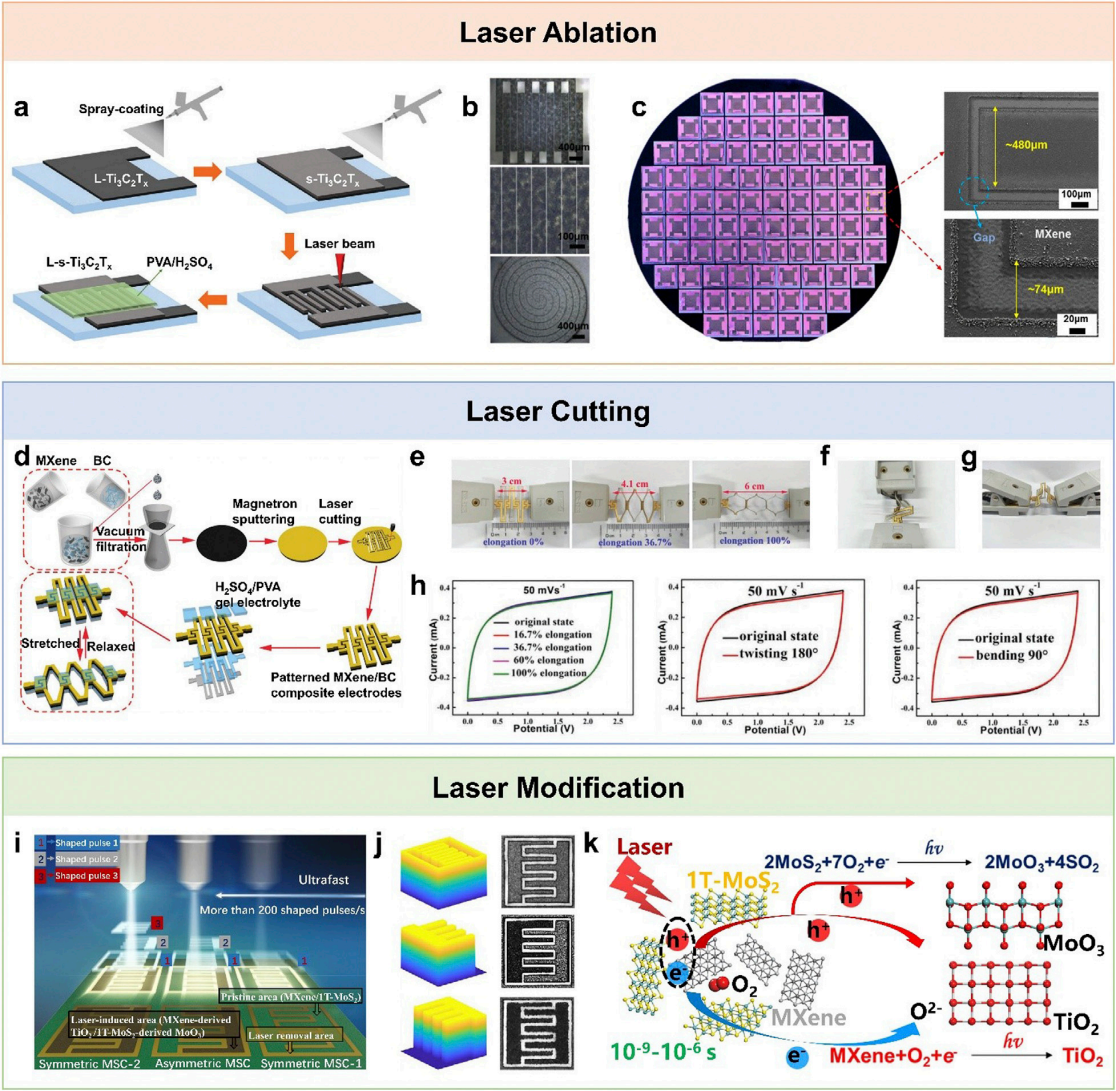
Fabrication of MXene-based planar supercapacitors using the laser technologies. **(a)** Fabrication process of planar MXene-based supercapacitors via laser ablation. The process began with spray-coating an L-Ti_3_C_2_T_x_ layer as the current collector, followed by deposition of an s-Ti_3_C_2_T_x_ electroactive film. The electrodes were then patterned by laser ablation, and a PVA/H_2_SO_4_ gel electrolyte was applied to complete the MSC device. Reproduced from (Peng et al., 2016) with permission of Royal Society of Chemistry. **(b)** Photographs of interdigital electrodes. Reproduced from (Li et al., 2020) with permission of WILEY-VCH Verlag GmbH and Co. KGaA, Weinheim. **(c)** Photograph of wafer-scale planar MXene-based supercapacitors. SEM images of MXene-based interdigitated electrodes and corresponding electrode gaps. Reproduced from (Huang et al., 2022) with permission of American Chemical Society. **(d)** Laser-cut fabrication process of stretchable, twistable, and bendable MXene-based supercapacitors. MXene/BC composite papers were fabricated via vacuum-assisted self-assembly and subsequently coated with an Au layer by magnetron sputtering. The papers were laser-cut into patterned electrodes to assemble MSC arrays, and schematic illustrations show their structural design and mechanical deformation under stretching. Photographs of an MXene-based supercapacitor array with four micro-supercapacitor units connected in series under **(e)** stretching, **(f)** twisting, and **(g)** bending. **(h)** Corresponding CV curves of the MXene-based supercapacitor array with four series-connected micro-supercapacitor units under stretching, twisting, and bending. Reproduced under the terms of the Creative Commons CC BY License (Jiao et al., 2019). Copyright 2019, The Authors, Published by WILEY-VCH Verlag GmbH and Co. KGaA, Weinheim. **(i)** Spatial Light Modulator-based rapid laser patterning modification method for MXene-based supercapacitors. **(j)** Light field patterns of various shapes and corresponding optical micrographs of different types of micro-supercapacitors fabricated via this process. **(k)** Mechanism of photo-induced chemical synthesis. Reproduced under the terms of the Creative Commons Attribution 4.0 International License (Yuan et al., 2023). Copyright 2023, The Authors, Published by Springer Nature.

For example, Peng et al., reported the fabrication of all-MXene solid-state MSCs (Peng et al., 2016). As shown in Figure 1a, large-size Ti_3_C_2_T_x_ flakes were first spray-coated as current collectors, followed by small-size Ti_3_C_2_T_x_ flakes as the electroactive layer. The interdigitated electrodes were then defined via direct laser ablation. Li et al. further advanced this approach using femtosecond laser ablation (Figure 1b), producing ultra-narrow electrode gaps of approximately 10 µm with minimal thermal damage to the MXene film (Li et al., 2020). The ultrashort pulses also enabled synchronous double-sided etching on transparent PET substrates. This process allows arbitrary on-substrate and through-substrate connections. It further supports high-density integration and modular series/parallel configurations to adjust the device voltage. This demonstrates the versatility of laser ablation for compact device integration while maintaining high electrochemical activity.

Notably, Huang et al. achieved Ti_3_C_2_T_x_ MSCs with an ultrahigh volumetric energy density of ∼75 mWh cm^-3^ and a working voltage of 1.2 V (Huang et al., 2022). In that study, the combination of acetone-modified MXene inks, natural sedimentation to suppress flake restacking, and precise laser ablation produced uniform interdigitated electrodes (Figure 1c). These studies highlight the advantages of laser ablation. It provides maskless, rapid, and flexible patterning. It also supports the scalable fabrication of MXene-based planar MSCs.

## 3 Laser cutting technologies

Laser cutting offers a straightforward and versatile approach for fabricating MXene-based MSCs, particularly suited for flexible and stretchable devices. The general process begins with preparing an MXene film on a flexible substrate. Typical substrates include paper, PET, and freestanding composite films. Common deposition techniques include vacuum filtration, slurry coating, and solution casting. A laser is then used to cut the MXene film into interdigitated electrode patterns, where the cut regions serve as separators and the remaining MXene film functions as both the active material and the current collector.

Kurra et al. demonstrated MXene-on-paper MSCs by Meyer-rod coating Ti_3_C_2_ films onto commercial printing paper, followed by rapid laser machining (Kurra et al., 2016). This method produced areal energy and power densities comparable to state-of-the-art paper-based devices. A representative example is the kirigami-patterned MXene/bacterial cellulose (BC) composite paper for all-solid-state stretchable MSC arrays (Jiao et al., 2019). In this work, freestanding MXene/BC composite paper with enhanced mechanical strength and reduced flake restacking was first prepared through a simple all-solution papermaking process (Figure 1d). Laser cutting was then employed to create kirigami interdigitated patterns, generating MSC arrays that were highly stretchable, bendable, and twistable without significant performance loss (Figures 1e-g). The resulting devices exhibited an areal capacitance of 111.5 mF cm^-2^ and retained electrochemical stability under 100% tensile strain, 180° twisting, and 90° bending (Figures 1h).

Other studies further highlighted the versatility of laser cutting. Chen et al. developed a laser-assisted “paste-tear” method on PET substrates, enabling the scalable fabrication of flexible MSC arrays with high areal capacitance (241 mF cm^-2^) and robust mechanical stability (Chen et al., 2022). Together, these studies show that laser cutting not only simplifies device fabrication but also enables the production of MSC arrays with excellent mechanical adaptability. These arrays can reliably withstand stretching, bending, and twisting.

## 4 Laser modification technologies

Laser modification technologies provide an effective strategy to enhance the performance of MXene-based planar MSCs by locally altering the physicochemical properties of pre-formed MXene films. Unlike laser ablation or cutting, which remove material, laser modification selectively irradiates the MXene film to induce localized photothermal or photochemical effects. These effects generate micro-/nanopores, partially oxidize and restructure MXene sheets, and thereby improve ion transport while increasing the accessible surface area. Reported variations in performance arise from the type of MXene precursors, the use of composites such as graphene oxide or bacterial cellulose, and the chosen laser parameters.

Tang et al. employed ultrafast laser writing to mitigate the restacking of Ti_3_C_2_T_x_ films, producing mesoporous channels that enhanced high-rate electrochemical performance (Tang et al., 2020). A representative example is the laser maskless fast patterning of multitype MSCs (Yuan et al., 2023). As shown in Figure 1i, femtosecond laser pulses were temporally and spatially shaped. These pulses were combined with a spatial light modulator. This approach enabled ultrafast and maskless fabrication of both symmetric and asymmetric MXene-based MSCs. By employing spatially multiplexed light fields, multiple MSC patterns as small as 10 × 10 μm^2^ can be produced simultaneously (Figure 1j). The mechanism involves selective removal and localized thermal oxidation of MXene/MoS_2_ to form Ti-rich and Mo-rich oxide nanoparticles, greatly improving processing efficiency and scalability for device integration (Figure 1k).

Other studies further demonstrate the versatility of laser modification. Deshmukh et al. reported picosecond laser-induced MXene-functionalized graphene nanoarchitectures (Deshmukh et al., 2023). In this process, Ti–O–C covalent bonds were formed *in situ*. This bonding yielded flexible MSCs with ultrahigh areal capacitance and long cycle life. Zhu et al. extended this concept to three-dimensional MXene-rGO architectures (Zhu et al., 2024), exploiting laser-triggered dimensional transformation and interlayer gas release to achieve high areal energy density with tunable interlayer spacing. Overall, laser modification technologies not only enhance the intrinsic electrochemical properties of MXene films but also enable scalable, rapid, and maskless fabrication of planar MSCs with high energy density. In particular, spatial light modulator-based multi-light-field processing represents a critical advance for high-throughput manufacturing of multitype MSCs.

## 5 Conclusion and outlook

Laser-based fabrication offers powerful, versatile routes for developing MXene-based planar MSCs. Laser ablation enables maskless patterning of interdigitated electrodes for compact on-chip MSCs. Laser cutting excels on flexible substrates, producing stretchable, bendable, and twistable MSC arrays with exceptional mechanical adaptability. Laser modification leverages localized photothermal and photochemical effects to tailor microstructure and surface chemistry, significantly improving ion transport and energy storage performance. Toward real-world applications, laser-fabricated MXene-based MSCs offer significant opportunities for industrial translation. Their compatibility with wearable electronics, portable power modules, and IoT devices highlights the potential to bridge laboratory advances with practical commercialization.
